# Creating Extraordinary From Ordinary: High Resource Efficiency of Underdog Entrepreneurs and Its Mechanism

**DOI:** 10.3389/fpsyg.2022.851356

**Published:** 2022-03-16

**Authors:** Hong-Ming Zhu, Xiong-Hui Xiao, Yanzhao Tang

**Affiliations:** ^1^School of Management, Xiamen University, Xiamen, China; ^2^Business School, Sun Yat-sen University, Guangzhou, China

**Keywords:** resource efficiency, underdog entrepreneurs, underdog effect, person-environment fit theory, psychosocial explanation, implicit theory, industry context, business environment

## Abstract

Existing theory has not documented the potential benefits of facing the challenges of underdog entrepreneurs, who may succeed unexpectedly. This research explains why, and under what circumstances, the underdog status of entrepreneurs can promote entrepreneurial success rather than just hinder it. We predict that the underdog effect has the potential to boost entrepreneurial resource efficiency when entrepreneurs hold an incremental (vs. entity) theory, enter a low-barrier (vs. high-barrier) industry, and are in a favorable (vs. unfavorable) business environment. Study 1 provides support for the positive relationship between underdog status and resource efficiency through an ordinary least squares (OLS) regression analysis, which is accompanied by a moderating effect of the implicit theory, industry context, and business environment. The data was obtained from two nationwide surveys. By extending a qualitative comparative analysis (QCA) of multiple case studies, Study 2 reveals support for a synergistic effect of the above factors. Our research results examine the assumption that perceiving underdog status is detrimental and offer meaningful insights into why and when underdog entrepreneurs have good performance in entrepreneurial resource efficiency. We provide a psychological and behavioral explanation for the underdog effect, extending the underdog effect theory to the field of entrepreneurship for the first time from the perspective of the actors. Finally, theoretical contributions and practical implications are discussed by indicating the limitations of the research.

## Introduction

Entrepreneurship is considered more inclusive than the general labor market. The heroic entrepreneur or ideal entrepreneur (Ahl, 2006) with outstanding personal characteristics and privileged resource endowments competes in the same arena with underdog entrepreneurs who have economic, sociocultural, cognitive, and physical disadvantages (Miller and Breton-Miller, 2017; Baron et al., 2018; Morgan, 2019). An underdog is defined in the Webster International English dictionary as “a predicted loser in a struggle or contest,” which means the expected loser in the competition, that is, the player who is not favored by the public. The underdog effect, which is a concern in academic research, refers to the mechanism effect that drives competitors in a weak position to win instead. This phenomenon is common in political elections, sporting events, film awards, and other contexts (Paharia et al., 2011; Nurmohamed, 2020). In entrepreneurship research, underdog entrepreneurs refer to entrepreneurs who are faced with unavoidable and difficult to change challenges in economy, social culture, cognition, body, or emotion (Miller and Breton-Miller, 2017), such as disabled entrepreneurs (Jammaers and Zanoni, 2020b; Martin and Honig, 2020), student entrepreneurs (Ahsan et al., 2018), veterans entrepreneurs (Heinz et al., 2017), refugee entrepreneurs (Bizri, 2017; Jiang et al., 2021), ethnic minority entrepreneurs (Neville et al., 2018), ADHD entrepreneurs (Wiklund et al., 2017), etc.

Although there is no lack of inspiring counterattack stories of underdog entrepreneurs in the media, they are more likely to experience entrepreneurial failure, and their organizations often experience low growth, limited innovation, and poor performance (Faggio and Silva, 2014; Mühlböck et al., 2017; Nikiforou et al., 2019; Assenova, 2020; Wierenga, 2020). On the surface, the underdogs may not be suitable for entrepreneurship because their contribution to economic growth and job creation is minimal (Wennekers et al., 2005), and they may experience more significant life setbacks due to entrepreneurial failure (Caliendo and Kritikos, 2010; Mühlböck et al., 2017). However, some scholars have put forward reflections on entrepreneurship research that concretizes entrepreneurship as an economic phenomenon for wealth production and ignores the multiple values that entrepreneurship can produce (Dodd et al., 2021). Therefore, repositioning entrepreneurship, paying attention to a wider range of entrepreneurial groups, including underdogs, and focusing on diversified values other than economic value have been suggested as a new research agenda (Miller and Breton-Miller, 2017; Wiklund et al., 2019b; Dodd et al., 2021).

It is worth noting that underdog entrepreneurs do not start their businesses to seek opportunities and growth (Anderson and Obeng, 2017; Garcia-Lorenzo et al., 2017; Wiklund et al., 2019b) but for other reasons, including salary substitution, being their own boss (Douglas, 2013), dealing with unemployment, escaping discrimination in the labor market, and integrating into society (De Clercq and Honig, 2011). Meanwhile, underdog entrepreneurs have substantial disadvantages in resource endowment (Mühlböck et al., 2017; Baron et al., 2018) and a large gap in resource investment compared with other entrepreneurs. Therefore, we cannot comprehensively and accurately evaluate the achievements and value of underdog entrepreneurship by ignoring their unique entrepreneurial motivations and substantial disadvantages in resource endowments. Moreover, ignoring these aspects may conceal the huge welfare value (Verduijn et al., 2014) and social value of circular frugality (Dodd et al., 2021) in underdog entrepreneurship.

This analysis indicates that exploring why and under what circumstances the underdog status may stimulate unique driving forces and generate unique entrepreneurial value is an interesting and significant issue that we focus on in this article. Research on the underdog effect (Paharia et al., 2011; Bothner et al., 2012; Nurmohamed, 2020) indicates that the underdog status may be a source of power. Entrepreneurs with an underdog status may work harder (Nurmohamed, 2020), approach problems differently (Miller and Breton-Miller, 2017), have a strong desire to change their current situation (Genicot and Ray, 2017), have sufficient experience to deal with uncertainties and frustrations (Kish-Gephart and Campbell, 2015), and be familiar with the needs of disadvantaged people (Wierenga, 2020). Therefore, we argue that although the underdog status means that they face additional multiple obstacles in entrepreneurship (Jammaers and Zanoni, 2020b), underdog entrepreneurs can still achieve high resource efficiency by exploiting the underdog effect. Unlike previous studies that emphasized the firm level and economic outcomes, this paper uses the individual resource efficiency of entrepreneurs to evaluate the entrepreneurial outcomes, reflecting the overall efficiency of the individual’s resource investment in entrepreneurship transformed into entrepreneurial returns. Among them, the entrepreneurial return adopts the latest definition of entrepreneurial success, including monetary and non-monetary achievements (Hatak and Zhou, 2021), while resource investment includes the financial capital and human capital investment (Lofstrom et al., 2014). In addition, based on the person-environment fit theory, this paper constructs and examines the assumptions and theories of how the individual implicit theory, industrial environment, and institutional environment strengthen or weaken the positive effects of the underdog status on resource efficiency. This paper finds that underdog entrepreneurs achieve higher resource efficiency due to their underdog status when they hold an incremental theory, start a business in a low-barrier industry, and are in a favorable business environment. The extended research based on the qualitative comparative analysis (QCA) of multiple cases further finds that the synergy of the implicit theory and industry selection play a decisive role in the resource efficiency of underdog entrepreneurs. Overall, this research explains why and under what circumstances the underdog status can promote entrepreneurial success rather than just hinder it.

This paper makes several contributions to the existing literature. First, we measure entrepreneurial performance from a new perspective of resource efficiency and contribute to the evaluation of the entrepreneurial results of underdog entrepreneurs. Most existing evaluations of entrepreneurial performance are based on pure results and focus more on economic aspects without considering the difference in the resource input (Wiklund et al., 2019a; Dodd et al., 2021). This article proposes resource efficiency as a measure of entrepreneurial performance to narrow this gap and reflect the value of entrepreneurship from a new perspective. This approach takes into account the differences in the entrepreneurs’ resource endowments and the unique reasons for entrepreneurship and considers the multi-target results of economic and non-economic returns. This approach considers all entrepreneurial costs and consequences, resulting in a more fair and comprehensive evaluation of the value of underdog entrepreneurship. This article finds that underdog entrepreneurs have high resource efficiency and achieve considerable entrepreneurial returns with limited investment. This finding challenges the assumption that underdog entrepreneurs always perform poorly. Second, this paper contributes to research on the underdog effect. Current research on this effect has mainly focused on sports, politics, and marketing and has been primarily based on the observer’s perspective (Nurmohamed, 2020). This paper extends the underdog effect theory to the field of entrepreneurship from the perspective of the actors. It explains how the underdog effect is generated and promotes entrepreneurial performance from the perspective of behavior and psychology. Revealing this underdog effect may be the key to revealing the survival skills of underdog entrepreneurs in extremely harsh environments. Third, this paper reveals the boundary conditions of the underdog status affecting resource efficiency. Existing research has shown that integrating an opportunity and resource perspective can improve entrepreneurial performance in individuals with high levels of vocational interests, grit, and regulated emotions (Arco-Tirado et al., 2019; Bergner, 2020; Li et al., 2021). This research shifts the benefit of a group of underdog entrepreneurs from internal psychological capital to external environmental-related factors. Specifically, we extend the personal-environment fit theory to our analysis and find that the relationship between underdog status and entrepreneurial performance is different when underdog entrepreneurs hold different implicit theories or are in different environments.

In addition, this research provides practical guidance for helping underdog entrepreneurs to start their own businesses. It helps entrepreneurs understand the impact of their disadvantages on entrepreneurial performance, encouraging them to turn disadvantages into advantages to choose a suitable entrepreneurial environment. This research provides valuable information for improving government policies. It can also inspire the public to look at the benefits of underdog entrepreneurs from a new perspective to provide more informal support for them.

## Theoretical Background and Hypotheses Development

### Underdog Entrepreneurs and Resource Efficiency

Both academic research and practical reports have not shown enough interest in entrepreneurs from disadvantaged groups (Dy, 2020), which are called “silent minorities” (Morgan, 2019). Miller and Breton-Miller (2017) developed a model of challenge-based entrepreneurship to reflect this phenomenon and called these individuals underdog entrepreneurs. The term underdog entrepreneur does not have a clear and consistent definition, but it is often used to represent entrepreneurs among disadvantaged people who cannot find jobs, face societal marginalization, or have low social status (Miller and Breton-Miller, 2017; Baron et al., 2018). In previous studies, different types of entrepreneurs were classified as underdog entrepreneurs, including extremely poor people (Lee et al., 2018; Xiong et al., 2018; Shepherd et al., 2021), ADHD (Patel et al., 2021; Yu et al., 2021), the physically handicapped (Renko et al., 2016; Jammaers and Zanoni, 2020b), veterans (Hope and Mackin, 2011), seniors (Kautonen et al., 2011; Maalaoui et al., 2019), the unemployed (Caliendo and Kritikos, 2010), immigrants (Clark et al., 2016; Dabić et al., 2020), ethnic minorities (Carter et al., 2015), and refugees (Shepherd et al., 2020).

Scholars have conducted extensive research on the benefits of positive individual characteristics to entrepreneurial performance. A number of studies on the resource endowment of entrepreneurs have shown that economic capital, human capital, and social capital have a positive impact on entrepreneurial success. Research on underdog entrepreneurs also holds a similar view, i.e., due to the inherent disadvantages, underdog entrepreneurs are often at a disadvantage in the competition, resulting in an adverse impact on entrepreneurial performance. First, the underdog status will lead to a lack of access to resources, making it difficult to obtain the resources that new enterprises rely on for survival and development (Baron et al., 2018). In the context of limited resources, resource efficiency was defined as the effort performance to achieve the goal of growth by maximizing the use of resources (Koh et al., 2016). Underdog entrepreneurs, such as student entrepreneurs, may choose entrepreneurial education to cultivate human capital (Salamzadeh et al., 2013, 2014; Dana et al., 2021), as well as use limited resources to launch Minimum Viable Products (MVP) and implement business innovation to become financially effective and resource efficient (Salamzadeh et al., 2017; Lopez et al., 2019). In addition, underdog entrepreneurs are easily threatened by stereotypes. They are often regarded as lacking in capacity, being inefficient, and lacking innovation (Jammaers and Zanoni, 2020a,b), which not only makes it difficult to obtain the recognition of resource gatekeepers but also reduces their self-efficacy, fosters anxiety (Nurmohamed, 2020), and reduces entrepreneurial performance. Therefore, underdog entrepreneurs are engaged in a competition with great disparity in strength, and they seem to be doomed to lose.

However, stories of successful counterattacks by underdog entrepreneurs have been reported in the literature and in sports and politics (Vandello et al., 2007; Paharia et al., 2011). Scholars have called the phenomenon of observers sympathizing with and supporting people who are in an underdog status in the competition as the observer’s underdog effect (Vandello et al., 2007; Goldschmied and Vandello, 2009; Paharia et al., 2011). Similarly, the phenomenon that the underdog status inspires an unexpected outstanding performance of the individual is referred to as the actor’s underdog effect (Nurmohamed, 2020), which is consistent with the underdog theory of entrepreneurship, which states that adversity contributes to the propensity for entrepreneurship (Miller and Breton-Miller, 2017). This study focuses on the actor’s underdog effect. We believe that this underdog effect may also exist in the entrepreneurial process, leading to high resource efficiency. Revealing this effect may be the key to revealing the survival skills of underdog entrepreneurs in extremely harsh environments. Entrepreneurship scholars have largely adopted psychological and behavioral perspectives to explain individual differences in entrepreneurship (Lerner et al., 2021). Here, we also provide a psychological and behavioral explanation for the underdog effect.

#### Psychological Explanations

Nietzsche (1977) once said: “What does not kill me makes me stronger.” The early adversity experience and various disadvantages of underdog entrepreneurs result in at least three psychological motivations that can promote resource efficiency. First, underdog entrepreneurs believe they have “nothing left to lose.” The current situation of underdog entrepreneurs is full of difficulties and challenges, and they have a strong desire to escape this condition. The theory of aspirations and poverty traps (Dalton et al., 2016; Chivers, 2017) shows that extremely poor people feel they have “nothing left to lose”; thus, they will seize every opportunity to escape their current situation, even viewing entrepreneurship as the only way out of poverty (Chivers, 2017; Genicot and Ray, 2017). Therefore, they are more likely to be eager and have a strong ambition to pursue an opportunity to create wealth (Dencker et al., 2021). They show great determination and courage to break the caldrons and sink the boats, and they are full of passion and excitement (Nurmohamed, 2020). These people are less likely to be afraid of failure, enabling them to act decisively. Second, underdog entrepreneurs want to “prove others wrong.” They have experienced various adversities in their lives. Negation and frustration are common emotions (Miller and Breton-Miller, 2017). They have long accumulated strong psychological resources and skills to deal with frustration and denial. Starting a business is a formidable challenge for any entrepreneur, but underdog entrepreneurs are more likely to be questioned by others and are expected to lose (Paharia et al., 2011; Jun et al., 2015; Nurmohamed, 2020). According to the psychological reactance theory, this will stimulate their strong desire to prove that other people’s views are wrong (Nurmohamed, 2020), resulting in heightened cognitive and affective engagement and producing potential positive results (Nurmohamed, 2020). Third, underdog entrepreneurs believe that “happiness lies in contentment.” They are excluded from the traditional labor market, and entrepreneurship can increase their dignity and respect (Miller and Breton-Miller, 2017); thus, they are more likely to be satisfied, forming a positive feedback cycle of self-enhancement (Sedikides and Gregg, 2008).

#### Behavioral Explanations

In order to survive early adversity, underdog entrepreneurs are forced to develop cognition and skills that enable them to take different entrepreneurial actions (Lofstrom et al., 2014; Miller and Breton-Miller, 2017; Baron et al., 2018), which is conducive to improving their resource efficiency. First, underdog entrepreneurs are more eager for opportunities, and they will seize all potential opportunities, even menial work that others are unwilling to do (Cobbinah and Chinyamurindi, 2018). At the same time, they have strong risk aversion and typically pursue short-term opportunities and implement imitation and small-scale entrepreneurship; therefore, they can often obtain considerable benefits at a lower cost (Douglas, 2013). Second, due to the lack of resources, underdog entrepreneurs are more diligent and cherish resources more, and they are able to maximize the development and utilization of limited resources creatively (Sarkar, 2018; Michaelis et al., 2020). For example, Pansera and Sarkar (2016) found that “resource-scarce entrepreneurs craft solutions that are environmentally friendly, with low overall ownership costs, and use locally available material.” Third, underdog entrepreneurs are not bound by resource traps; thus, they tend to focus more on improving products and services and treat key audiences such as customers with more care. Underdog entrepreneurs may have better knowledge of people’s demands at the bottom of the pyramid (Cuervo-Cazurra and Genc, 2008; Hall et al., 2012). According to the findings of Piff et al. (2010), entrepreneurs from the lower classes have a higher level of prosocial behavior. They have less but give more, which helps them maintain a good relationship with stakeholders and improve their own well-being. Fourth, underdog entrepreneurs are more self-disciplined and more able to endure hardships. In addition, external disadvantages and early adversity helps them understand that a person needs to rely on others to achieve their goals (Rucker et al., 2018). Therefore, they are often more willing to seek help and to care for and help others, and they place greater emphasis on social relations and cooperation (Abele and Wojciszke, 2007; Diekman et al., 2010). Fifth, underdog entrepreneurs have a high level of determination and perseverance in the face of obstacles. They are more persistent and more indomitable (Paharia et al., 2011) and typically find unusual approaches to solving problems. Therefore we hypothesize:

**Hypothesis 1:** The underdog status of entrepreneurs strengthens the underdog effect and enables entrepreneurs to obtain higher resource efficiency.

### The Moderating Role of the Implicit Theory, Industry Context, and Business Environment

Person–environment fit is defined as the level of compatibility between individuals and their working environment (Edwards and Cooper, 1990). It is a powerful predictor of individual or organizational outcomes (van Vianen, 2018). Entities can achieve better outcomes (e.g., job satisfaction, task performance) when the attributes of a person and environment are compatible. Entrepreneurship is a product of self and the circumstances (Dodd et al., 2021). An entrepreneur creates and extracts values from an environment (Anderson, 2000). Underdog entrepreneurs have different backgrounds and personal characteristics than other entrepreneurs; thus, there is a need to match the entrepreneurial circumstances to the underdog entrepreneur to achieve optimal outcomes. Based on the person–environment fit theory, this article focuses on three aspects, i.e., the individual implicit theory, the industry context, and the business environment, to evaluate the impact of the environment and an individual’s understanding of himself/herself and the environment on the relationship between the underdog status and entrepreneurial achievements.

#### Implicit Theory

Implicit theory is a belief system of individuals to understand the social world they live in. It can be divided into two types: entity theory and incremental theory (Dweck et al., 1993, 1995). The entity theorist believes that human attributes (personality, intelligence, and morality) are fixed. When interpreting events or behaviors, they tend to rely on these fixed characteristics. In contrast, the incremental theorist believes that these characteristics are dynamic and malleable. When interpreting events or behaviors, people evaluate more specific factors, such as intentions, goals, or emotions. The implicit theory held by an individual has a profound influence on his or her behavior (Dweck et al., 1993, 1995), affecting the individual’s responses to challenges and setbacks and leading to different interpretations of events and different expectations for the future (Dweck, 2008; Davis et al., 2010). Due to external disadvantages, underdog entrepreneurs face more challenges than other entrepreneurs. When confronted with tough situations and setbacks, underdog entrepreneurs who are entity theorists are more likely to attribute a bad performance to their talent and fixed traits, resulting in helplessness. In comparison, incremental theorists prefer to update their cognition based on the event itself and the relevant factors, such as a lack of experience or effort or unfamiliarity with risk control, which can be improved in follow-up activities (Dweck et al., 1993; Hong et al., 1999; Molden and Dweck, 2006). Therefore, compared with entity theory, incremental theory is more likely to help underdog entrepreneurs escape the trap of being disadvantaged, overcome external constraints, and actively respond to setbacks and challenges in the entrepreneurial process, resulting in the underdog effect. Due to rapid change and complex dynamic outsider expectations, it is crucial for underdog entrepreneurs to believe in the incremental theory and obtain valuable information to develop dynamic capabilities for entrepreneurial survival and development. Therefore, we formally propose:

**Hypothesis 2:** The entrepreneurs’ implicit theory moderates the underdog effect toward entrepreneurial resource efficiency: underdog entrepreneurs who hold an incremental (vs. entity) theory are more likely to generate high entrepreneurial resource efficiency.

#### Industry Context

Based on the work of Lofstrom et al. (2014), we compared the impact of two different industry contexts, namely, high-barrier industries and low-barrier industries. The former have higher requirements for the entrepreneurs’ financial capital investment or knowledge and technology, whereas the latter have relatively low requirements for both aspects. We believe that underdog entrepreneurs can fully exploit the underdog effect and achieve higher resource efficiency in low-barrier industries. There are two main reasons: first, underdog entrepreneurs have higher autonomy regarding working hours, workplaces, working methods, and working conditions in low-barrier industries. Thus, they are more likely to enjoy entrepreneurship (Douglas, 2013; Wiklund et al., 2019a). When they start a business in a high-barrier industry, their work is more complex and more stressful, which may force them to work harder. As a result, they are more likely to be frustrated and sacrifice work for fun and life enjoyment due to the high workload (Lofstrom et al., 2014). In general, entrepreneurship in low-barrier industries can provide beneficial psychological rewards to underdog entrepreneurs, but it does not need to bear the psychological costs of more complex enterprise bosses. On the other hand, several psychological and behavioral advantages associated with the underdog status, such as courage, diligence, persistence, proactively seeking help, finding ways to solve problems, and a deep understanding of the needs of specific groups (Miller and Breton-Miller, 2017), are an important force to promote entrepreneurial achievements in low-barrier industries with relatively low requirements for financial capital, knowledge, and technology. However, in high-barrier industries that require high levels of financial capital, knowledge, and technology, these psychological and behavioral advantages are difficult to offset the disadvantages of underdog entrepreneurs in terms of resource endowment, knowledge, and technology (Baron et al., 2018). Based on the above analysis, we propose the following hypothesis.

**Hypothesis 3**: The specific industry context moderates the underdog effect toward entrepreneurial resource efficiency: starting businesses in low-barrier industries (vs. high-barrier industries) is more likely to generate high entrepreneurial resource efficiency of underdog entrepreneurship.

#### Business Environment

The business environment is a comprehensive ecosystem of the external environment that companies face when they engage in entrepreneurship, innovation, financing, investment, and other activities. It is the institutional prerequisite for entrepreneurs to engage in entrepreneurial activities and determines whether new ventures can obtain fair access to key sources (Lim et al., 2010). The Doing Business Report issued by the World Bank is an authoritative analysis assessing the regulations and environment that encourage efficiency and support the freedom to do business (World Bank, 2020). Start-ups generally face strong resource constraints, and underdog entrepreneurs face additional obstacles. Their savings and education levels are low, they lack specific industry knowledge and experience (Assenova, 2020), they are often discriminated against by resource gatekeepers, and they have difficulty obtaining resources (Baron et al., 2018; Jammaers and Zanoni, 2020b). Some underdog entrepreneurs are forced to use bribery and other informal means to obtain resources (Baron et al., 2018). A favorable business environment can stop people from pursuing dangerous brinkmanship, such as bribery. Furthermore, the underdog entrepreneur can obtain social support from the business environment, such as tax deductions, rent relief in the early stage, and product purchase support. Underdog entrepreneurs receive more empathy and compassion than other entrepreneurs (Jun et al., 2015; He et al., 2020). Due to the inclusive environment, there is less social exclusion and fewer resource constraints (Hall et al., 2012; Sutter et al., 2019). Both the economic and non-economic performance can be improved, unlike in an unfavorable business environment. Therefore, the following hypothesis is proposed:

**Hypothesis 4**: The business environment moderates the relationship between the underdog status and entrepreneurial resource efficiency: starting businesses in a favorable business environment (vs. unfavorable) is more likely to generate high entrepreneurial resource efficiency of underdog entrepreneurship.

## Empirical Analysis

To gain a better understanding of the antecedents of the high resource efficiency achieved by underdog entrepreneurs, two studies are conducted for empirical analysis. Study 1 tests the proposed hypothesis through the ordinary least squares (OLS) regression analysis, which is one of the most widely used methods in quantitative research. Second hand data from two nationwide surveys are used. We believe that using two databases from two different sources with different investigation purposes can reduce the sample selection and measurement bias and provide more robust evidence for research conclusions. Overall, Study 1 is conducted to identify the correlations between underdog status and resource efficiency, as well as the moderating effect of the implicit theory, industry context, and business environment.

Then, we used QCA to investigate the drivers and configurations of the high resource efficiency, which Study 1 could not answer. QCA is used to compare and analyze cases using Boolean logic and algebra to examine the synergistic effect of the interaction between multiple factors on specific phenomena (Rihoux and Ragin, 2008). Rather than just discovering correlations between independent and dependent variables, QCA finds patterns of elements that contribute to a given conclusion (Mikalef and Pateli, 2017). Specifically, the fuzzy set QCA (fsQCA) method (a subcategory of QCA) was applied to examine the interaction effect of the underdog status, implicit theory, industry context, and business environment on entrepreneurial resource efficiency. The primary distinction between fsQCA and other QCA approaches is that fsQCA permits outcome and predictor variables to be on a continuous scale rather than a binary scale (Mikalef and Pateli, 2017). We analyzed the configuration of the forward cases and reverse cases simultaneously.

### Study 1: Empirical Study Based on Two National Surveys

#### Data and Sample

The data for this study comes from two large-scale panel surveys in China, i.e., the China Labor-force Dynamic Survey (CLDS) conducted by the Social Science Survey Centre of Sun Yat-sen University and the Enterprise Survey for Innovation and Entrepreneurship in China (ESIEC) conducted by Peking University. We applied for these two national panel surveys from the official agencies for academic research use. The official websites are http://css.sysu.edu.cn and https://opendata.pku.edu.cn/. The former database is used for the empirical analysis, and the latter is used for robustness testing. The CLDS focuses on the current situation and changes in China’s labor force. Since 2012, a survey has been conducted every 2 years, covering many research topics, such as education, work, health, social participation, and economic activities. This study uses CLDS data from 2014 and 2016. The independent variables, moderating variables, control variables, and entrepreneurial investment-related data for calculating the rate of entrepreneurial resource efficiency originate from the 2014 individual and community data sets. The data of the entrepreneurial returns for calculating entrepreneurial resource efficiency are from the 2016 data set. The CLDS database has the following advantages. First, the sample is nationally representative, covering 29 provinces and cities in China. The survey objects are workers in sample households. Second, the database provides longitudinal tracking data, which is more conducive to analyzing entrepreneurial investment, the dynamic relationship between returns, and their influencing factors. The CLDS has more than 16,000 samples, including more than 2,000 entrepreneur samples, which we can get the target sample from the occupational types. After matching the 2014 and 2016 data to extract tracking samples and excluding data with missing values for the independent variables and dependent variables, we screened out 581 entrepreneurs^1^. The ESIEC is a special survey that reflects the micro-level status of Chinese companies’ innovation and entrepreneurship. From the data of this national survey conducted by different agency, we can get a robust result. It has been conducted annually since 2017. The survey content includes the entrepreneurs’ entrepreneurial history, corporate creation process, basic corporate information, corporate innovation, inter-enterprise relationships, and business operations. The database contains 1410 entrepreneur samples. After excluding samples with missing values of the independent variable and dependent variable, 436 entrepreneur samples were obtained. We used the Stata 16 software to process the OLS regression analysis. We believe that using two databases from two different sources with different investigation purposes reduces the sample size and measurement bias and provides stronger evidence for research conclusions.

#### Variables

##### Dependent Variable

###### Entrepreneurial Resource Efficiency

Following the research by Fernández-Serrano et al. (2017), data envelopment analysis (DEA) is used to calculate the overall efficiency of the entrepreneurs’ multiple inputs and returns. The input includes two factors, namely, human capital and economic capital, representing the core resource input of the entrepreneur. The output includes three factors, i.e., entrepreneurial income, job satisfaction, and life well-being, representing the monetary and non-monetary returns obtained by the entrepreneur. See Table 1 for the measurement method of each factor.

**TABLE 1:** Variable measurement design of Study 1.

No.	Variable	Variable meaning	Variable operation
1	*Underdog_s*∼*s*	Underdog status	When there is any one of the five major disadvantages, the value is 1, indicating that there is an underdog status, otherwise the value is 0.
1.1	*Poor_SES*	Poor socio-economic status	When the individual’s socioeconomic status index is lower than the lower 25th quantile, the value is 1, otherwise the value is 0. According to Li (2005), the socio-economic status index = 10.868 + 3.496*years of education + 0.589*average monthly income (100 yuan).
1.2	*PCE_limitations*	Physical cognitive and emotional defects	When there are physical, cognitive, or emotional limitations, the value is 1, otherwise the value is 0.
1.3	*Lack_EKS*	Lack of experience, knowledge, and skills	If individual have been unemployed before starting a business, the value is 1, otherwise the value is 0.
1.4	*Dis_social_network*	Lack of social network and social support	Question item: “Locally, how many friends/acquaintances do you have close relationships that you can get their support and help?” When the answer is 0, the value is 1, otherwise the value is 0.
1.5	*Dis_location*	Unfavorable geographical location	It is measured by the population density of the community, if it is lower than the average, it is taken as 1, and otherwise it is taken as 0.
2	*DEA_Sc*∼*e*	Entrepreneurial resource efficiency	The DEA method is used to calculate the comprehensive efficiency of multi-input and multi-return. The input includes two factors of human capital and economic capital, and the output includes three factors of material return and non-material return.
2.1	*Entre_investment_financial*	Economic capital investment	Question item: How much was the capital invested when your business started?
2.2	*Entre_investment_human*	Human capital investment	Measured by years of education
2.3	*Entre_income*	Entrepreneurial income	Operating income in 2015
2.4	*Wellbeing*	Life well-being	Question item: Generally speaking, do you think you are living a happy life? 1–5 means very unhappy-very happy.
2.5	*Job_satisfaction*	Job satisfaction	Question item: Please rate your overall satisfaction with your current job. 1–5 means very dissatisfied-very satisfied.
3	*Implicit_t*∼*y*	Implicit theory	Question item: Some people feel that they can choose their own life completely, while some people feel that they can’t do anything about what happened to them. How do you feel about your freedom to choose your life? Value 1–10, 1 means no option at all, 10 means great option.
4	*Industry_t*∼*d*	Industry context	When the financial capital barrier and human capital barrier are both low, the industry is regarded as a low-barrier industry, with a value of 1. When an industry has the characteristics of a high financial capital barrier or high human capital barrier, it is regarded as a high-barrier industry, and the value is 2.
5	*Business_e*∼*t*	Business environment	Question item: How many days did it take to start a business from application to obtaining a license? According to the answer converted into a score of 0–5, the longer the time, the lower the score.
6	*Gender*	Gender	If the gender is “male,” the value is 1, and if the gender is “female,” the value is 2.
7	*Age*	Ager	Respondent’s age
8	*Marriage*	Marriage	(1) Unmarried, (2) First marriage, (3) Remarried, (4) Divorced, (5) Widowed, (6) Cohabiting

##### Independent, Moderating, and Control Variables

###### Underdog Status

External disadvantages are crucial to revealing the underdog status (Paharia et al., 2011), referring to relatively large obstacles and few resources. Miller and Breton-Miller (2017) argue that underdog entrepreneurs confront economic, sociocultural, cognitive, and physical challenges. Mechanic and Tanner (2007) proposed four sources of vulnerability for vulnerable groups, including poverty and race, social networks and lack of social support, personal limitations, and physical location. Based on the work of these scholars, we put forward five types of external disadvantages that underdog entrepreneurs may face, including poor socio-economic status; physical cognitive and emotional defects; lack of experience, knowledge, and skills; lack of social network and social support; unfavorable geographical location. When entrepreneurs face disadvantages, they are considered to be in an underdog status, receiving a value of 1; otherwise, the value is 0. The specific measurement method of each disadvantage is shown in Table 1.

###### Implicit Theory

The following survey question is used in the CLDS regarding the moderating variable-implicit theory: “some people feel they can completely choose their own life, while others feel they cannot do anything about events affecting them. How free do you feel to choose your own life?”. The value range is 1–10; 1 means no choice, and 10 means many choices. The survey question in the ESIEC is “Please score the importance of different factors (talent, education, and effort) to personal success.” If the talent score is greater than the scores of education and effort, the person is an entity theorist, with a value of 0. If the scores are equal, the person is neutral, and the value is 1. If the talent score is less than the scores of education and effort, the person is an incremental theorist, with a value of 2.

###### Industry Context

For the industry context moderating variable, we follow the method of Lofstrom et al. (2014) and divide the entrepreneurial industry into high-barrier industries and low-barrier industries according to the requirements for financial capital, knowledge, and technology. There are two sub-barriers, namely, the financial capital barrier and the human capital barrier. When an industry has the characteristics of a high financial capital barrier or high human capital barrier, it is regarded as a high-barrier industry, and the value is 2. When the financial capital barrier and human capital barrier are both low, the industry is regarded as a low-barrier industry, with a value of 1. The financial capital sub-barrier is based on the average start-up investment of all entrepreneurs. Those higher than the average are regarded as facing a high financial capital barrier, and those below the average face a low financial capital barrier. The human capital sub-barrier is based on the average highest education of all entrepreneurs. Those higher than the average are considered to face a high human capital barrier, and those below the average are considered to face a low human capital barrier.

###### Business Environment

For the business environment moderating variable, we used the business environment indicator system based on the World Bank’s Doing Business Report, which has more than ten indicators, such as setting up enterprises, handling construction permits, and obtaining electricity. Based on the calculation method of the World Bank’s business environment index and existing other data, we used the following method to measure the business environment. The CLDS uses only the index of setting up enterprises; thus, this score is used as a proxy variable of the business environment. In the ESIEC, the business environment information is more comprehensive, including the evaluation of starting a business, construction permits, obtaining electricity, tax returns, government projects, and executing contracts. The existing data are used to determine the business environment based on the calculation method of the World Bank.

###### Control Variables

We selected demographic variables, such as age, gender, and marital status of the entrepreneurs as control variables.

#### Results

##### Descriptive Statistics

Table 2 lists the descriptive statistics and correlation results of all variables. The correlation coefficients are less than 0.5, indicating no significant multicollinearity.

**TABLE 2:** Descriptive statistics and correlation analysis.

Variable	Mean	*SD*	(1)	(2)	(3)	(4)
*(1) Gender*	1.4	0.49	1			
*(2) Age*	43.01	9.65	–0.101**	1		
*(3) Marriage*	2.08	0.59	0.004	0.078*	1	
*(4) Underdog_s*∼*s*	0.6	0.49	0.161***	0.123***	0.007	1
*(5) DEA_Sc*∼*e*	0.35	0.36	–0.055	0.314***	0.064	0.131***
*(6) Implicit_t*∼*y*	7.09	2.08	–0.053	0.047	0.05	–0.090**
*(7) Industry_t*∼*d*	1.16	0.37	–0.146***	–0.094**	0.035	–0.093**
*(8) Business_e*∼*t*	4.09	0.87	0.044	0.109	0.045	-0.066
Variable	Mean	*SD*	(5)	(6)	(7)	(8)
*(1) Gender*	1.4	0.49				
*(2) Age*	43.01	9.65				
*(3) Marriage*	2.08	0.59				
*(4) Underdog_s*∼*s*	0.6	0.49				
*(5) DEA_Sc*∼*e*	0.35	0.36	1			
*(6) Implicit_t*∼*y*	7.09	2.08	–0.024	1		
*(7) Industry_t*∼*d*	1.16	0.37	–0.188***	0.062	1	
*(8) Business_e*∼*t*	4.09	0.87	0.037	-0.028	-0.115	1

*, **, and *** denote statistical significance at the 10%, 5%, and 1% level.

Table 3 lists the *t*-test results of comparing the characteristics of underdog entrepreneurs and non-underdog entrepreneurs. The preliminary results show that the entrepreneurial resource efficiency of underdog entrepreneurs is significantly higher than that of non-underdog entrepreneurs. Although the entrepreneurial income of underdog entrepreneurs is significantly lower, the investment of resources is also significantly lower. However, there is no significant difference in life well-being and job satisfaction between the two types of entrepreneurs. This finding has special significance due to the status of underdog entrepreneurs.

**TABLE 3:** Characteristics of underdog entrepreneurs and non-underdog entrepreneurs.

Variable	Non-underdog entrepreneur	Underdog entrepreneur	MeanDiff
*Gender*	1.31	1.47	–0.161***
*Age*	41.56	43.99	–2.423***
*Marriage*	2.08	2.09	–0.009
*DEA_Sc*∼*e*	0.30	0.39	–0.095***
*Implicit_t*∼*y*	7.32	6.93	0.382**
*Industry_t*∼*d*	1.20	1.13	0.070**
*Business_e*∼*t*	4.16	4.04	0.12
*Invest_financial*	82000.00	54000.00	28000**
*Invest_human*	9.81	8.74	1.073***
*Entre_income*	53000.00	28000.00	25000***
*Wellbeing*	3.92	3.82	0.10
*Job_sati*∼*n*	3.60	3.54	0.06

** and *** denote statistical significance at the 5% and 1% level.

##### Hypotheses Testing

Table 4 reports the results of the relationship between the underdog status and the entrepreneurial resource efficiency and the test results of the moderating effect of the implicit theory, industry context, and business environment on the relationship. M2 is a main effect test. The results show that the underdog status and entrepreneurial resource efficiency are significantly positively correlated (β = 0.073, *p* < 0.05), indicating that the underdog status results in higher entrepreneurial resource efficiency. Hypothesis 1 is verified. M2 is a test of the moderating effect of the implicit theory. The results show that the coefficient of the interaction term between the underdog status and the implicit theory is significant and positive (β = 0.030, *p* < 0.05), demonstrating that entrepreneurial resource efficiency is higher for an underdog entrepreneur holding the incremental theory. M3 is a test of the moderating effect of the industry context. The results show that the coefficient of the interaction term between the underdog status and the industry context is significant and negative (β = –0.171, *p* < 0.05), i.e., the industry context weakens the positive effect of the underdog status on entrepreneurial resource efficiency. Therefore, underdog entrepreneurs in low-barrier industries have higher entrepreneurial resource efficiency than those in high-barrier industries. M4 examines the moderating effect of the business environment. The results show that the coefficient of the interaction term between the underdog status and the business environment is significant and negative (β = 0.094, *p* < 0.1), i.e., the business environment enhances the effect of the underdog status on the entrepreneurial resource efficiency. In other words, underdog entrepreneurs achieve higher entrepreneurial resource efficiency in a favorable business environment.

**TABLE 4:** Regression analysis results of underdog status, three moderating variables and entrepreneurial resource efficiency (CLDS data).

Variable	M1	M2	M3	M4
*Underdog_s*∼*s*	0.073**	–0.147	0.266***	–0.301
	(–0.03)	(0.108)	(0.092)	(0.236)
*Implicit_t*∼*y*		–0.026**		
		(0.012)		
*Underdog_s*∼*s* × *implicit_t*∼*y*		0.030**		
		(0.014)		
*Industry_t*∼*d*			–0.071	
			(0.055)	
*Underdog_s*∼*s* × *industry_t*∼*d*			–0.171**	
			(0.075)	
*Business_e*∼*t*				–0.044
				(0.040)
*Underdog_s*∼*s* × *business_e*∼*t*				0.094*
				(0.056)
*Gender*	–0.03	–0.031	–0.047	0.012
	(–0.03)	(0.029)	(0.029)	(0.049)
*Age*	0.011***	0.011***	0.010***	0.010***
	(0.000)	(0.001)	(0.001)	(0.003)
*Marriage*	0.024	0.026	0.028	0.005
	(–0.02)	(0.024)	(0.023)	(0.033)
*_cons*	–0.166*	0.012	–0.044	–0.021
	(–0.09)	(0.122)	(0.116)	(0.221)
*N*	581	575	581	165
*r2*	0.11	0.1177	0.1443	0.1295
*r2_a*	0.104	0.1084	0.1353	0.0964
*F*	17.851	12.63	16.13	3.92

*, **, and *** denote statistical significance at the 10%, 5%, and 1% level.

**FIGURE 1:**
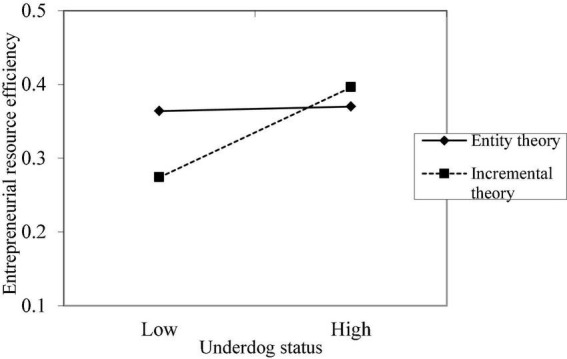
The moderating effect of implicit theory on the relationship between underdog status and entrepreneurial resource efficiency.

**FIGURE 2:**
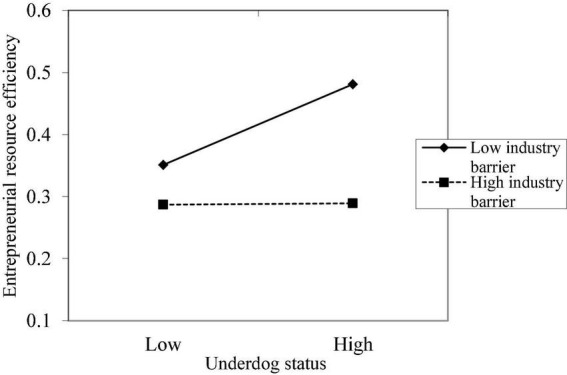
The moderating effect of industry context on the relationship between underdog status and entrepreneurial resource efficiency.

**FIGURE 3:**
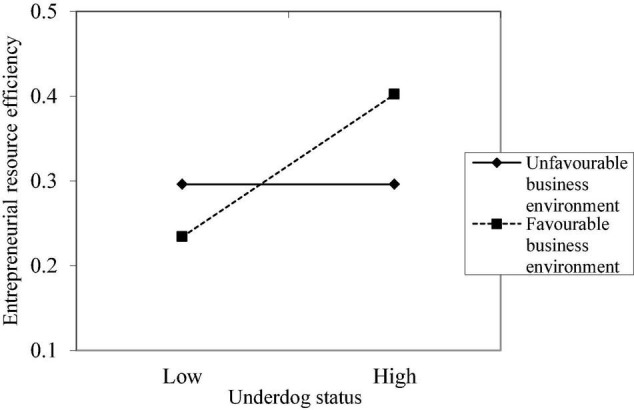
The moderating effect of business environment on the relationship between underdog status and entrepreneurial resource efficiency.

The graphs of the moderating effects of the implicit theory, industry context, and business environment on the relationship between the underdog status and entrepreneurial resource efficiency are shown in Figures 1–3, respectively. It is observed that the three variables have significant moderating effects on the relationship between the underdog status and entrepreneurial resource efficiency, indicating that Hypotheses 2, 3, and 4 are verified.

##### Robustness Check

We used the ESIEC data to test all the hypotheses of this study; the test results are shown in Table 5. M5 is the main effect test. The results show that the underdog status and the entrepreneurial resource efficiency are significantly positively correlated (β = 0.060, *p* < 0.01). Hypothesis H1 is verified again. M6 is a test of the moderating effect of the implicit theory. The results show that the coefficient of the interaction term between the underdog status and the implicit theory is significant and positive (β = 0.052, *p* < 0.05), i.e., there is a positive effect on the entrepreneurial resource efficiency if the underdog entrepreneur holds the incremental theory. Hypothesis 2 is verified again. M7 examines the moderating effect of the industry context. The results show that the coefficient of the interaction term between the underdog status and the industry context is significant and negative (β = –0.07, *p* < 0.1), i.e., the industry context weakens the positive effect of the underdog status on entrepreneurial resource efficiency, verifying Hypothesis 3. M8 examines the moderating effect of the business environment, and Hypothesis 4 is verified again (β = 0.039, *p* < 0.01). The test results show that all hypotheses are verified, demonstrating the robustness and reliability of the results of this study.

**TABLE 5:** Regression analysis results of underdog status, three moderating variables and entrepreneurial resource efficiency (ESIEC data).

Variable	M5	M6	M7	M8
*Underdog_s*∼*s*	0.060***	–0.028	0.156***	–0.267**
	(0.020)	(0.045)	(0.060)	(0.118)
*Implicit_t*∼*y*		–0.018		
		(0.020)		
*Underdog_s*∼*s* × *implicit_t*∼*y*		0.052**		
		(0.026)		
*Industry_t*∼*d*			–0.026	
			(0.030)	
*Underdog_s*∼*s* × *industry_t*∼*d*			–0.07*	
			(0.04)	
*Business_e*∼*t*				–0.014
				(0.010)
*Underdog_s*∼*s* × *business_e*∼*t*				0.039***
				(0.014)
*Gender*	0.091***	0.082***	0.095***	0.089***
	(0.025)	(0.025)	(0.025)	(0.026)
*Age*	0.003***	0.003***	0.003***	0.003***
	(0.001)	(0.001)	(0.001)	(0.001)
*Marriage*	0.000	0.000	0.000	0.000
	(0.000)	(0.000)	(0.000)	(0.000)
*_cons*	–0.055	0.010	–0.02	0.056
	(0.049)	(0.055)	(0.064)	(0.094)
*N*	436	424	436	435
*r2*	0.079	0.0701	0.108	0.102
*r2_a*	0.070	0.0568	0.095	0.089
*F*	9.190	5.24	8.610	8.070

*, **, and *** denote statistical significance at the 10%, 5%, and 1% level.

### Study 2: An Extended Case Study Based on Qualitative Comparative Analysis

QCA is used to compare and analyze cases using Boolean logic and algebra to examine the synergistic effect of the interaction between multiple factors on specific phenomena (Rihoux and Ragin, 2008). Thus, we used QCA to extend this research on underdog entrepreneurs and investigate the drivers and configurations of the high resource efficiency, which the above Study 1 could not answer. Specifically, the fuzzy set QCA (fsQCA) method (a subcategory of QCA) was applied to examine the interaction effect of the underdog status, implicit theory, industry context, and business environment on entrepreneurial resource efficiency. We analyzed the configuration of the forward cases and reverse cases simultaneously.

#### Data and Sample

The samples of this case study were obtained from the large-scale entrepreneurial competition launched by China Central Television called “Entrepreneurship Hero Meeting.” The program aims to support mass entrepreneurship and inspire innovation. It seeks entrepreneurial projects with growth potential, gathering heavyweight entrepreneurial mentors and professional investors. Abundant funds and resources are available to build a platform for entrepreneurs to propose their business ventures and realize their dreams.

The program was first released by entrepreneurs conducting a roadshow of entrepreneurial projects. Two on-site mentors were voting. If both votes were positive, the entrepreneur could enter the second round of negotiations with investors. If only one vote was positive, the live audience voted; the entrepreneur entered the second round when the support rate exceeded 70%. If both votes were negative, the entrepreneur had to leave. In the second round, entrepreneurs and investors interacted and exchanged questions to determine investment and financing intentions. In order to avoid external influences and case selection bias caused by external factors, such as the replacement of show hosts and entrepreneurial mentors, we selected all entrepreneurs of the show from December 25, 2015 to February 5, 2016 as study samples. Finally, 21 entrepreneurs were used as case samples (Table 6).

**TABLE 6:** Case samples of Study 2.

No.	Project name	Entrepreneur	Years in business	Age	Gender	Industry
1	LvJi Tour guide	Zang**	6	35	M	Travel software
2	Large character set application technology coding project	Wang**	9	65	M	Computer technology
3	WeiBang Smart sports bed	Wang**	7	34	M	Medical rehabilitation equipment
4	Internet + Bird Love Cheongsam	Cui**	15	39	M	Clothing
5	Youth League Club	Deng**	3	25	M	Software
6	TianYi Drone	Chen***	2	33	M	Drone
7	Smart car cover	Liang*	2	42	M	Car supplies
8	Net rush	Wu**	2	32	M	Community service
9	Gaokao Net	Bai*	0.5	27	M	Information service
10	Masu bao	Wu*	5	39	M	Information service
11	Xun Qiu APP	Hu*	1	28	M	Information service
12	JuMeiYi	Wang*	2	33	M	Information service
13	Micro-nano fiber	Zhao*	5	36	F	New material
14	Hao Mai MRM	Min**	3	34	M	Information service
15	YePu	Yan*	2	36	M	Hardware + software
16	Cloud Move house	Li**	2	39	M	Software
17	YouJi	Tian**	1	30	F	Software
18	3D Optical materials	Ke**	3	36	M	New material
19	Intelligent voice assistant	Tong*	2	23	M	Software
20	Bilingual Music Class	Ning**	1	34	F	Education service
21	ChaJia Mouthwashs	Zhao**	15	43	M	Life items

* denote omitted Chinese character.

#### Measures and Calibration

First, the “insider-outsider” coding method was used to determine the values of the underdog status, implicit theory, and industry context variables (Corbin and Strauss, 2008). One of our authors and a researcher that was not part of the author team coded the television program data into variable levels. Second, another author of this study checked the consistency and validity of the coding result, ensuring an in-depth and accurate understanding of the qualitative data. The underdog status, implicit theory, and industry context variables were codes the same as in Study 1 (0–1). The entrepreneurial resource efficiency was coded using a 5-point Likert scale, where 1 denotes very low entrepreneurial resource efficiency and 5 denotes very high entrepreneurial resource efficiency. Additionally, the data on the business environment was obtained from the “2017 China City Business Environment Report” released by the Academy of Greater Bay Area Studies. They match the city’s business environment index based on the entrepreneur’s location.

Unlike in quantitative research, the data in the fsQCA must be calibrated before analysis (Ragin, 2008; Schneider and Wagemann, 2012). We used 0.95, 0.5, and 0.05 as the thresholds for the underdog status, implicit theory, and industry context variables. We used 5.001, 3.001, and 1.001 as the thresholds for the entrepreneurial resource efficiency and 0.629, 0.594, and 0.423 for the business environment index, according to Ragin (2008).

#### Findings

##### Necessity Analysis

The results of the necessity of the conditions (Ragin, 2008) are listed in Table 7. It is found that there is no necessary condition for high efficiency and low efficiency since all consistency levels are lower than 0.9.

**TABLE 7:** Necessity analysis.

Causal condition	Consistency	Coverage	Causal condition	Consistency	Coverage
Underdog status	0.357	0.412	Industry context	0.828	0.415
∼Underdog status	0.719	0.434	∼Industry context	0.247	0.469
Implicit theory	0.771	0.500	Business environment	0.317	0.601
∼Implicit theory	0.305	0.312	∼Business environment	0.759	0.380

##### Sufficiency Analysis of the Conditional Configuration

Different from the necessary condition analysis, the sufficiency analysis of the configuration aims to reveal different configurations composed of multiple conditions leading to the same result. Schneider and Wagemann (2012) pointed out that the consistency level of determination sufficiency should not be less than 0.75, which was used in our research. The fsQCA analysis outputs three solutions with different levels of complexities, i.e., a complex solution, parsimonious solution, and intermediate solution. This research reports the intermediate solution, supplemented by the parsimonious solution, which is consistent with extant research (Fiss, 2011). The results of the sufficiency analysis are listed in Table 8.

**TABLE 8:** Conditional configuration sufficiency analysis.

Causal configuration	High entrepreneurial resource efficiency		Low entrepreneurial resource efficiency		
	1	2	3	4	5
Underdog status	●	⊗	⊗		●
Implicit theory	●	⊗	•	⊗	⊗
Industry context	⊗	•	⊗	•	●
Business environment		●	•	⊗	
Consistency	0.932	0.932	1.000	0.941	0.867
Raw coverage	0.257	0.211	0.120	0.364	0.251
Unique coverage	0.188	0.142	0.067	0.184	0.071
Solution consistency	0.921		0.909		
Solution coverage	0.399		0.509		

*Keep consistent with Fiss (2011), black circles indicate the presence of a condition, and circles with “ × ” indicate its absence. Large circles indicate core conditions; small ones, peripheral conditions. Blank spaces indicate “don’t care”.*

The five solutions indicate that the consistency level is higher than the acceptable minimum standard of 0.75 for the single solution (configuration) and the overall solution. The consistency of the high entrepreneurial resource efficiency is 0.921, and the coverage of the overall solution is 0.399, reaching the QCA consistency level in the organization and management field.

Configuration 1 and Configuration 5 have the underdog status as the core condition, representing two configurations in which entrepreneurs in an underdog status achieve high entrepreneurial resource efficiency and low entrepreneurial resource efficiency. Configuration 1 can be expressed as “*Underdog status* × *Implicit theory* × ∼*Industry context→High entrepreneurial resource efficiency*,” and Configuration 5 as “*Underdog status* × ∼*Implicit theory* × *Industry context→Low entrepreneurial resource efficiency.*” Specifically, “implicit theory” means that people hold the incremental theory of achievement, and “∼implicit theory” implies people hold the entity theory of achievement. “∼*Industry context*” indicates that the industry barrier is low. For underdog entrepreneurs, the synergy of the implicit theory and industry selection significantly affects entrepreneurial resource efficiency.

Configuration 2 and Configuration 3 have the non-underdog status as the core condition, representing the two configurations of high entrepreneurial resource efficiency and low entrepreneurial resource efficiency. Configuration 2 can be expressed as “∼*Underdog status* × ∼*Implicit theory* × *Business environment→High entrepreneurial resource efficiency*,” and Configuration 3 as “∼*Underdog status* × ∼*Industry context→Low entrepreneurial resource efficiency.*” These results demonstrate that industry choice is of utmost importance for non-underdog entrepreneurs.

In addition, Configuration 4 can be converted into “∼*Implicit theory* × ∼*Business environment→Low entrepreneurial resource efficiency.*” “∼Business environment” means an unfavorable business environment. The configuration implies that people who hold the entity theory of achievement in an unfavorable business environment cannot achieve high entrepreneurial efficiency.

In summary, underdog entrepreneurs should take advantage of the incremental theory of achievement and choose an appropriate fit industry. They should also utilize the business environment as non-underdog entrepreneurs do to achieve high resource efficiency and performance.

## Discussion

### Results Discussion

Underdog entrepreneurs are entrepreneurial groups that can be seen everywhere but are often ignored by academic research. They are usually forced to start a business because they have no better job options (Miller and Breton-Miller, 2017). As a result of their underdog status, these entrepreneurs face multiple obstacles in the entrepreneurial process, and they are more likely to experience low entrepreneurial performance than other entrepreneurs (Mühlböck et al., 2017; Assenova, 2020). However, underdog status may also be a source of power, allowing underdog entrepreneurs to attain unexpectedly high levels of success. Using resource efficiency as a yardstick for measuring entrepreneurial performance, we investigated why and under what conditions underdog status may stimulate efforts and promote performance.

Our analysis shows that underdog entrepreneurs can achieve high resource efficiency by exploiting the underdog effect. On one hand, as a result of their underdog status, entrepreneurs have accumulated strong psychological resources and skills during their early challenging experiences. They have “nothing left to lose,” showing determination and courage to change their situation. They are not easily affected by negation but will prove themselves when there is doubt. On the other hand, underdog status shapes underdog entrepreneurs’ unique behavior characteristics. For example, they are more eager for opportunities, cherish resources, work harder and persevere, and have a unique understanding of the needs of customers.

The emergence of this “underdog effect” is affected by individual implicit theory, industry context, and business environment. Compared with entity theory, the incremental theory is more likely to help underdog entrepreneurs break the vulnerability trap, overcome external constraints, and actively deal with setbacks and challenges in the process of entrepreneurship. In the industrial context, entrepreneurship in low-threshold industries can further strengthen the positive impact between underdog status and entrepreneurial resource efficiency because a series of psychological and behavioral advantages inspired by underdog status, such as courage, diligence, persistence, actively seeking help, trying to solve problems, and deeply understanding the needs of specific groups. In low-threshold industries with relatively low requirements for capital and technical knowledge, it will become an important force to promote entrepreneurial achievements. However, in high-threshold industries, the above psychological and behavioral advantages are difficult to offset the disadvantages of underdog entrepreneurs in resource endowment and technical knowledge level, resulting in the limited role of underdog status in promoting entrepreneurial achievements. Finally, a favorable business environment can enable underdog entrepreneurs to more effectively obtain resources from the business environment ecosystem, reduce institutional transaction costs, alleviate financing constraints, boost entrepreneurial confidence, help solve the difficulties encountered by underdog entrepreneurs, and make the underdog effect play better.

In addition, based on the QCA study of underdog entrepreneurs, it can be found that the incremental style holders of the implicit theory accompanied by a low-threshold industry requirement can stimulate the underdog effect to the maximum. It can be seen that for underdog entrepreneurs, the synergy of implicit theory and industry choice plays a decisive role in entrepreneurial resource efficiency. For non-underdog entrepreneurs, industry selection is very important.

In conclusion, we achieved results that offer fresh theoretical insights as well as policy consequences by using resource efficiency as the primary research variable and investigating how underdog entrepreneurs achieve unexpectedly high resource efficiency.

### Theoretical Contributions

Our research provides the following theoretical contributions. First, this article deepens the understanding of the entrepreneurial achievements of underdog entrepreneurs. Studies on entrepreneurial performance typically emphasize firm-level outcomes, such as growth and performance (Wiklund et al., 2019a). These studies did not consider the background of entrepreneurs and differences in resource input since businesses operated by underdog entrepreneurs are often considered to have low growth, low innovation, and low performance (Faggio and Silva, 2014; Mühlböck et al., 2017; Nikiforou et al., 2019; Assenova, 2020). Our research focuses on exploring the positive aspects that a disadvantaged background brings to entrepreneurs, and we conduct a fairer and more comprehensive evaluation of the value of underdog entrepreneurs by evaluating entrepreneurial resource efficiency, which is a comprehensive measure of entrepreneurial performance. The differences in resource endowments and the unique reasons for individual entrepreneurship are considered, integrating the multi-target results of economic and non-economic outcomes. This study finds that underdog entrepreneurs have high entrepreneurial resource efficiency that is achieved with limited investment. This result challenges the assumption that underdog entrepreneurs are always underperforming.

Second, this article supplements existing research on the underdog effect. Current research on this topic has mainly concentrated on sports, politics, and marketing and has been based on the observer’s perspective, reflecting the phenomenon that observers sympathize with and support people in an underdog status in competition (Vandello et al., 2007; Goldschmied and Vandello, 2009; Paharia et al., 2011). Based on the perspective of the actor (Nurmohamed, 2020), this article expands the research on the underdog effect to the field of entrepreneurship and analyses how the disadvantaged background of entrepreneurs contributes to an unexpected outstanding performance. Moreover, this article provides a psychological and behavioral explanation of the underdog effect and explores the unique psychological motivation and distinctive skills and behaviors of entrepreneurs in the underdog status. Revealing this underdog effect may be the key to revealing the survival skills of underdog entrepreneurs in extremely harsh environments, providing a new insight into how the underdog status of entrepreneurs affects entrepreneurial results.

Third, this research contributes to the related theories of the psychological process and the person-environment fit theory by revealing the effect of the boundary conditions of the underdog status on entrepreneurial performance (Edwards and Cooper, 1990; van Vianen, 2018; Stappers and Andries, 2021). This study found different relationships between the underdog status and entrepreneurial performance when underdog entrepreneurs held different implicit theories or were in different industry and business environments, significantly improving our understanding of the influence of the underdog status on entrepreneurial performance. Specifically, holding the incremental theory, being in a low-barrier industrial environment, and having a favorable business environment promote the underdog effect and strengthen the positive impact of the underdog status on entrepreneurial resource efficiency. Furthermore, this article also finds that the most significant underdog effect is observed when the entrepreneur holds the incremental theory and establishes a business in a low-barrier industry.

### Practical Implications

This research explores the relationship between the underdog status of entrepreneurs and entrepreneurial achievements, explores the psychological and behavioral advantages of underdog entrepreneurs and the characteristics of high entrepreneurial resource efficiency, and reveals which factors promote the underdog effect. The results have the following practical implications:

First, from the perspective of underdog entrepreneurs and underdog groups who intend to enter entrepreneurship, this research reveals the potential positive effects of their disadvantaged background on entrepreneurship, guiding them to turn their disadvantages into advantages to achieve their entrepreneurial goals. In addition, the analysis of the boundary of the underdog effect helps potential entrepreneurs to evaluate the industrial environment and their own characteristics when starting a business to choose a favorable business location and actively analyze and respond to difficulties and setbacks.

Second, from the perspective of administrative management, this research reveals the necessity and special significance of providing targeted support to underdog entrepreneurs. The unique background and differences of underdog entrepreneurs should be considered in formulating policies supporting entrepreneurs. In addition, in previous poverty alleviation policies, policymakers emphasized the transition from “blood transfusion” to “hemopoiesis.” The underdog effects revealed in this study can help formulate relevant policies.

Third, from the perspective of public opinion, this research reveals that underdog entrepreneurs do not always perform poorly. They have high entrepreneurial resource efficiency, which can inspire the public to look at the benefits of underdog entrepreneurs from a new perspective, be more inclusive and encouraged, and provide more informal support for underdog entrepreneurs to start their own businesses.

### Limitations and Future Research Directions

Our study has the following limitations, which we plan to address in follow-up studies. First, our research proves the existence of the underdog effect through empirical research and theoretically provides a psychological and behavioral explanation of the underdog effect. However, due to data limitations, we could not determine the specific mediating mechanism between the disadvantaged background and the success of underdog entrepreneurs. Future research could help to establish theories that explain the complicated influence of vulnerability and peculiarity brought on by disadvantaged origins on entrepreneurship, as well as reveal how underdog entrepreneurs exploit their oddity to turn disadvantages into advantages. Case studies can be used to investigate the specific mechanism of the formation of the vulnerable effect, and experimental design can be used to assess the influence of various situational circumstances in terms of research methodologies.

Second, this research evaluates underdog entrepreneurs’ entrepreneurial outcomes from a novel perspective of resource efficiency, challenging the assumption that disadvantaged entrepreneurs are always underperforming. However, this just reflects one part of the underdog entrepreneurial value assessment, and the DEA method adopted in this paper has certain difficulties in practical application. The causes, purpose, and ideals of entrepreneurship must all be considered when evaluating entrepreneurial value. Entrepreneurship should be viewed as a widely used social instrument that may help underdog entrepreneurs alter their own lives, families, communities, and other settings, as well as the well-being, empowerment, and freedom of underdog groups and even larger issues. As a result, the future study can pay more attention to disadvantaged entrepreneurs’ diverse entrepreneurial motivations, appreciate the diversified value of entrepreneurship, and comprehend and evaluate their entrepreneurial contribution from a new perspective.

Third, this research demonstrates that underdog and non-underdog entrepreneurs have different success logics and behavioral patterns. More discoveries, however, must be made through more in-depth comparative research of underdog and non-underdog entrepreneurs. Conducting research on the unique characteristics of underdog entrepreneur groups and comparing entrepreneurial behavior to that of other entrepreneur groups will be able to make unique contributions to entrepreneurial research. This is a fascinating and significant subject that deserves further investigation. Future research might look into the distinctive behavior of underdog entrepreneurs and how they differ from non-underdog entrepreneurs using a variety of methodologies, such as case studies.

## Conclusion

Underdog entrepreneurship is an interesting research subject as well as a significant occurrence. As our research shows, underdog entrepreneurs have special entrepreneurial background, heterogeneous entrepreneurial motivation, and exhibit distinct entrepreneurial behavior and performance characteristics. Focusing on the heterogeneity of this group can make a unique contribution to the existing entrepreneurial research. Currently, mainstream entrepreneurship research is paying more attention to underdog entrepreneurs, but there is still a lot of room for research and plenty of chances. As a result, we encourage other scholars to follow in our footsteps, explore how underdog status affects entrepreneurs’ motivation, behavior, and performance in greater depth. This will aid scholars in better understanding entrepreneurship heterogeneity, enhance entrepreneurship research, and provide useful insight for entrepreneurial practice.

## Data Availability Statement

Publicly available datasets were analyzed in this study. This data can be found here: http://css.sysu.edu.cn.

## Author Contributions

H-MZ was in charge of the research design and the implementation of Study 1. X-HX was in charge of the implementation of Study 2. YT made substantial intellectual contributions to the conception of the study and draft of the manuscript. All the authors read and approved the final version of the manuscript.

## Conflict of Interest

The authors declare that the research was conducted in the absence of any commercial or financial relationships that could be construed as a potential conflict of interest.

## Publisher’s Note

All claims expressed in this article are solely those of the authors and do not necessarily represent those of their affiliated organizations, or those of the publisher, the editors and the reviewers. Any product that may be evaluated in this article, or claim that may be made by its manufacturer, is not guaranteed or endorsed by the publisher.

## Footnotes

1 Since some moderating variables had missing values, the number of samples was slightly different for the moderating effect analysis.
